# Frontline Science: TNF‐α and GM‐CSF1 priming augments the role of SOS1/2 in driving activation of Ras, PI3K‐γ, and neutrophil proinflammatory responses

**DOI:** 10.1002/JLB.2HI0918-359RR

**Published:** 2019-02-05

**Authors:** Sabine Suire, Fernando C. Baltanas, Anne Segonds‐Pichon, Keith Davidson, Eugenio Santos, Phillip T. Hawkins, Len R. Stephens

**Affiliations:** ^1^ Inositide Laboratory The Babraham Institute Cambridge UK; ^2^ Centro de Investigación del Cáncer—Instituto de Biología Molecular y Celular del Cáncer (CSIC‐ Universitad de Salamanca) and CiberONC Salamanca Spain; ^3^ Bioinformatics Group The Babraham Institute Cambridge UK

**Keywords:** PI3K, RasGEF, SOS

## Abstract

Circulating neutrophils are, by necessity, quiescent and relatively unresponsive to acute stimuli. In regions of inflammation, mediators can prime neutrophils to react to acute stimuli with stronger proinflammatory, pathogen‐killing responses. In neutrophils G protein‐coupled receptor (GPCR)‐driven proinflammatory responses, such as reactive oxygen species (ROS) formation and accumulation of the key intracellular messenger phosphatidylinositol (3,4,5)‐trisphosphate (PIP_3_), are highly dependent on PI3K‐γ, a Ras‐GTP, and Gβγ coincidence detector. In unprimed cells, the major GPCR‐triggered activator of Ras is the Ras guanine nucleotide exchange factor (GEF), Ras guanine nucleotide releasing protein 4 (RasGRP4). Although priming is known to increase GPCR–PIP_3_ signaling, the mechanisms underlying this augmentation remain unclear. We used genetically modified mice to address the role of the 2 RasGEFs, RasGRP4 and son of sevenless (SOS)1/2, in neutrophil priming. We found that following GM‐CSF/TNFα priming, RasGRP4 had only a minor role in the enhanced responses. In contrast, SOS1/2 acquired a substantial role in ROS formation, PIP_3_ accumulation, and ERK activation in primed cells. These results suggest that SOS1/2 signaling plays a key role in determining the responsiveness of neutrophils in regions of inflammation.

## Graphical abstract

SOS1/2 signalling plays a key role in determining the responsiveness of neutrophils in regions of inflammation.

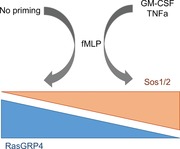

**Abbreviations**
Gβγ: heterotrimeric G‐protein βγ subunit dimer
GEF: guanine nucleotide exchange factor
GPCR: G protein‐coupled receptor
NET: neutrophil extracellular trap
PIP_3_: phosphatidylinositol (3,4,5)‐trisphosphate
PLC: phospholipase C
RBD: Ras‐binding domain
ROS: reactive oxygen species
RTK: receptor tyrosine kinase
SOS: son of sevenless
RasGRP4: Ras guanine nucleotide releasing protein 4
TAN: tumor‐associated neutrophil
WT: wild type

## 1 INTRODUCTION

Neutrophils constitute around 60% of the circulating white blood cells in humans. They play a critical role in immune defence against invading pathogens and have a major role in inflammation. They respond quickly by migrating to the sites of inflammation and neutralize potentially injurious agents by phagocytosis and by releasing degradative enzymes, neutrophil extracellular traps (NETs) and reactive oxygen species (ROS). These actions are toxic for pathogens but can also damage local tissue and drive many inflammatory diseases such as rheumatoid arthritis or acute respiratory distress syndrome. It is therefore very important to restrict their activation in time and space.^1^ It is believed that “priming” is one of the key mechanisms that bring this safeguard, as only by prior exposure to a priming agent (chemoattractant, proinflammatory cytokine, or TLR agonist) neutrophils can maximally respond to a subsequent challenge with a ligand such as *N*‐fMLP.^2^

In recent years, neutrophils have been found infiltrating many types of tumors and it is becoming clearer that tumor‐associated neutrophils (TANs) play a role in malignant disease.^3^ Two types of TAN have been described with antagonistic affects (pro‐tumor or anti‐tumor), which are determined by factors expressed by the stromal cells or the tumor itself. Given the presence and important roles for TNF‐α and GM‐CSF in the tumor microenvironment and the host response to cancer,^4^ it is very likely that the changes in neutrophil signaling and function induced by these ligands, and the process of priming, shape tumor progression.

Class I PI3Ks are responsible for receptor‐stimulated production of the phospholipid phosphatidylinositol 3,4,5‐trisphosphate (PI(3,4,5)P_3_, also known as PIP_3_). Class I PI3Ks are subdivided into class IA (PI3K‐α, PI3K‐β, and PI3K‐δ) and IB (PI3K‐γ), based on the properties of their regulatory subunits. The class IA PI3Ks are characteristically activated by receptor tyrosine kinase (RTK)‐based mechanisms involving binding of the SH2 domains in their regulatory subunits to phosphotyrosine residues within YXXM recognition sequences.^5^ PI3K‐γ is abundant in myeloid cells, particularly in neutrophils. It is a heterodimer comprising of a catalytic subunit, p110‐γ, and a regulatory subunit, p84 or p101.^6^ Genetic deletion of p110‐γ revealed that it is required for G protein‐coupled receptor (GPCR)‐stimulated PIP_3_ accumulation, PKB activation, ROS formation, and chemokinesis.^7^, ^8^, ^9^ PI3K‐γ is primarily regulated by heterotrimeric G‐protein βγ subunit dimer (Gβγ) subunits that bind directly to both the catalytic and regulatory subunits and activate the complex.^10^, ^11^ The other important regulator of PI3K‐γ (and class IA PI3Ks) is GTP‐bound Ras that can bind to, and activate, p110‐γ directly via its Ras‐binding domain (RBD).^12^, ^13^, ^14^ Unprimed mouse neutrophils expressing endogenous, Ras‐insensitive, but Gβγ‐sensitive, p110‐γ (p110γ^DASAA/DASAA^), have a similar phenotype to unprimed p110‐γ^−/−^ neutrophils.^15^ The Ras‐guanine nucleotide exchange factor (GEF) responsible for the GPCR‐stimulated activation of Ras in unprimed neutrophils is the Ras guanine nucleotide‐releasing protein‐4 (RasGRP4).^16^ RasGRP4 is activated by GPCRs via phospholipase C (PLC) β2/β3‐generated 1,2‐diacylglycerol (DAG). Hence, these results reveal full activation of PI3K‐γ in unprimed neutrophils requires Gβγ‐stimulation of PI3K‐γ directly and, indirectly, via PLCβ2/β3/DAG/RasGRP4/GTP‐Ras. Interestingly, although the interaction of GTP‐Ras with the RBD of p110γ is required for full GPCR stimulation of PI3K‐γ, activation of Ras alone, in the absence of GPCR stimulation, cannot activate PI3K‐γ in vivo. This is probably because Ras signals are not sufficient to activate PI3K‐γ and combine synergistically with inputs from Gβγs to determine PI3K‐γ activity.

The SOS proteins are an evolutionarily conserved family of RasGEFs. In mammals, there are 2 members, SOS1 (ubiquitously expressed and essential for mouse development) and SOS2. They can be activated by a number of mechanisms but are best known for their ability to be activated by RTKs via their interaction with GRB2, an SH2 domain‐containing protein that can bind RTKs directly or via a GRB2/SHC/RTK complex. GM‐CSF has been shown to drive tyrosine phosphorylation of SHC, its association with GRB2/SOS, and activation of Ras.^17^ SOS proteins can also be activated by GTP‐Ras in a potential positive feedback loop.^18^ This mechanism has been argued to be important in generating sustained, bimodal activation of Ras in lymphocytes and is based on receptor (TCR or BCR) stimulation of RasGRP1 achieving a threshold activation of Ras at which point SOS‐mediated positive‐feedback kicks‐in, generating a sustained, strong response.^19^

Neutrophils can be primed by a wide range of proinflammatory mediators and by a variety of mechanisms and time scales, including: regulated changes in the surface expression or binding properties of receptors, increased phosphorylation of NADPH oxidase components, and changed transcription and expression of many genes, particularly those encoding cytokines or chemokines.^20^ A defining feature of the priming process is that it leads to a dramatic increase in responses to a subsequent, and usually different, ligand suggesting that the mechanisms are not based on “additivity” or a common mechanism of action in the process. Given the central role of PI3K‐γ and PIP_3_ signaling in neutrophil responses, it seems likely that priming agents would target this pathway to modulate neutrophil responsiveness and there is evidence that supports this concept. Some neutrophil priming agents can stimulate PIP_3_ accumulation directly (e.g., GM‐CSF and GPCR‐ligands, such as fMLP, C5a)^21^, ^22^ and hence this creates a complex background in which to answer this question. TNF‐α, however, cannot increase PIP_3_ or GTP‐Ras levels in isolated neutrophils, but can prime granulocytes. The mechanism appeared to be dependent on a TNF‐α‐elicited, large increase in PIP_3_ accumulation after relatively prolonged (1–2 min) stimulation with the 2^0^ agonist. Importantly, priming with TNF‐α did not increase signaling via the closely related PLCβ2/β3 pathway indicating these events were not driven by increased GPCR signaling.^23^, ^24^ The mechanism by which priming elicited an increase in sustained, fMLP‐stimulated PIP_3_ accumulation is unclear; it appears to involve augmentation of class IA PI3K‐β/δ activity, but is also dependent on PI3K‐γ activity, leading to the idea that there is sequential activation of PI3K‐γ and PI3K‐β/δ.^24^

Mixtures of TNF‐α and GM‐CSF are known to be amongst the most powerful priming agents for both human and mouse neutrophils, and in this study, we have aimed to understand the mechanism by which a mixture of TNF‐α and GM‐CSF, which does not significantly increase PIP_3_ accumulation in isolation, substantially augments PIP_3_ accumulation and ROS formation in response to a subsequent dose of fMLP.

## 2 MATERIALS AND METHODS

### 2.1 Reagents and antibodies

All materials used were of the lowest endotoxin level available and were purchased from Sigma (UK) unless stated otherwise. The antibodies used for Western blots were commercially available: anti‐SOS1 (1:1000; Santa Cruz, Dallas, USA), anti SOS2 (1:1000; Santa Cruz), anti‐P‐PKB‐S473 (1:2000; Cell Signaling, Danvers, MA, USA); anti‐P‐p42/44 MAPK (T202/Y204) (1:2000; Cell Signaling), anti‐p47phox (1:4000; Upstate, Merck‐Millipore, UK), and anti‐β‐actin (1:10,000; Sigma). fMLP and PMA were from Sigma. Murine GM‐CSF was from Peprotech (London, UK) and murine TNF‐α from RnDSystems (Minneapolis, USA).

Internal standards for lipid analysis, 1‐heptadecanoyl‐2‐hexadecanoyl‐*sn*‐glycero‐3‐(phosphoinositol‐3,4,5‐trisphosphate) (C17:0/C16:0‐PIP_3_, as a hepta‐sodium salt) and C17:0/C16:0‐PI, were synthesized at the Babraham Institute. All chemicals and solution were of analytical reagent grade.

### 2.2 Mice

The PLCβ2^–/–^ x PLCβ3^–/–^mice^7^ (generously provided by D. Wu, Yale university, USA), the RasGRP4^–/–^mice,^16^ the p110‐γ mice^8^ (generously provided by M. Wymann, Basel, Switzerland), and SOS1/2 DKO (SOS1^fl‐Cre/fl‐Cre^/SOS 2^–/–^and their control (SOS1^+‐Cre/+‐Cre^/SOS 2^+/+^) mice^25^ (generously provided by E. Santos, Salamanca, Spain) have been previously described. In the case of the latter mice, to obtain a complete deletion of SOS1, the mice were fed for 5 days on a soya‐free diet and then for 10 days on a tamoxifen‐containing chow diet (Harlan, Teklad TAM 400/creER). The removal of SOS1 was monitored by immunoblotting. In all experiments, mice were compared with appropriate age‐ and strain‐matched wild‐type (WT) controls. All of the mice used in experiments were kept under specific pathogen free conditions in the Transgenics/Import units at the Babraham Institute. This work was performed under Home Office Project license PPL 70/8100.

### 2.3 Purification of mouse neutrophils

Murine neutrophils were isolated at room temperature from bone marrow of femurs and tibias using Percoll gradients (55% and 62%). The red blood cells contaminants were lysed (131 mM NH_4_Cl, 5 mM KCl, 0.8 mM Na_2_HPO_4_, 0.2 mM KH_2_PO_4_, 1 mM MgCl_2_, 0.3 mM MgSO_4_, 1.5 mM CaCl_2_ 13.4 mM NaHCO_3_, and 5.5 mM glucose) and the purity, assessed by flow cytometer, was typically between 65–85% neutrophils. Once counted and their purification established, the neutrophils were stimulated straight away (no priming condition) or primed for 1 h at 37°C in the presence of mouse TNF‐α (500U/ml) and GM‐CSF (2 or 0.2 ng/ml) or incubated for 1 h without any priming reagents (mock priming). To stop fMLP stimulations, after a defined time, 900 µl ice‐cold PBS (immunoblots and Ras activation assays) or 800 µl of ice‐cold 1 M HCl (PIP_3_ assays) were added to the incubations.

### 2.4 Immunoblots

The protein extracts were separated via SDS‐PAGE in 10% polyacrylamide gels and were transferred overnight onto PDVF membranes. After blocking and incubating with relevant primary and secondary, HRP‐labeled, antibodies, the membranes were incubated with ECL reagents (GE Healthcare) and exposed to light‐sensitive film. Protein levels were quantified by 2D densitometry using Aida Image Analyzer software v3.27.

### 2.5 ROS production

It was measured by chemiluminescence using a luminol‐based assay in polystyrene 96‐well plates by a Berthold Microlumat Plus luminometer (Berthold Technologies). At the end of their priming period, the neutrophils (0.5 × 10^6^) were incubated with luminol (150 µM) and HRP (18.75 U/ml) for 3 min at 37°C and their stimulation either with fMLP or PMA took place straight away following with the recording of the luminescence emission.

### 2.6 Ras activation assays

To measure the activation of Ras, neutrophils were stimulated while in suspension (4 × 10^6^ per condition) then rapidly diluted with cold PBS, sedimented by brief centrifugation (total time ∼10 s), aspirated and solubilized into ice‐cold lysis buffer. The lysates were centrifuged (13,500 × *g*, 10 min, 4°C) and the supernatants mixed with 4× SDS‐PAGE sample buffer. Ras pull‐down assays were performed using GST‐Raf‐RBD as previously described.^16^

### 2.7 Quantification of phosphoinositides

PIP_3_ in mouse neutrophils was quantified by mass spectrometry.^26^ Aliquots of neutrophil suspensions (153 µl, 0.5 × 10^6^) were stimulated with fMLP (17µl, 10 µM final concentration) or vehicle alone. After the appropriate time of stimulation, the incubations were quenched with 800 µl of 1 M HCl, centrifuged (12,000 rpm, 4°C, 5 min), and aspirated and then the pellets were stored at –80°C until processed. The pellets were resuspended in 920 µl of primary extraction solvent (MeOH, CHCl3, 1 M HCl, 484:242:23.55) and 2 internal standards were then added to correct any variations in recovery: D6‐1‐steroeoyl‐2‐arachidonyl‐DAG (10 pg) and C16:0/C17:0‐PIP_3_ (1 ng). CHCl_3_ (725 µl) and 2 M HCl (170 µl) were added, and the resulting 2 phases^27^ were separated by centrifugation (2000 × *g*, room temperature, 5 min). The organic, lower phase was then washed twice with an organic buffer (CHCl_3_, methanol, 0.01 M HCl, 24:12:9). The lower phase, now containing the PIP_2_ and PIP_3_, was derivatized using TMS‐diazomethane and washed twice with acid‐free organic buffer (CHCl3, Methanol, H_2_O, 24:12:9). Once dried under N_2,_ it is resuspended in 100 µl of methanol:water (9:1) and sonicated. The lipids were resolved by in‐line HPLC and analyzed by mass spectrometry, as described previously^26^; values for endogenous C18/C20:4 PIP_2_ and PIP_3_ were corrected for recovery of the C16:0/C17:0‐PIP_3_ internal standard_._

### 2.8 Statistics

Neutrophils were isolated from at least 2 mice (of the same genotype) and pooled prior to conducting experiments. Depending on the number of comparisons, two‐tailed *t*‐tests or ANOVAs followed by Holm–Sidak's multiple comparisons tests were used. When departure from normality was observed, data were log transformed prior to the analyses. Differences were considered significant *P*‐value < 0.05.

## 3 RESULTS

### 3.1 PLCβ2/β3, PI3K‐γ, and Ras‐activation of PI3K‐γ are required for fMLP‐stimulated ROS formation in both unprimed and primed mouse neutrophils

**Figure 1:**
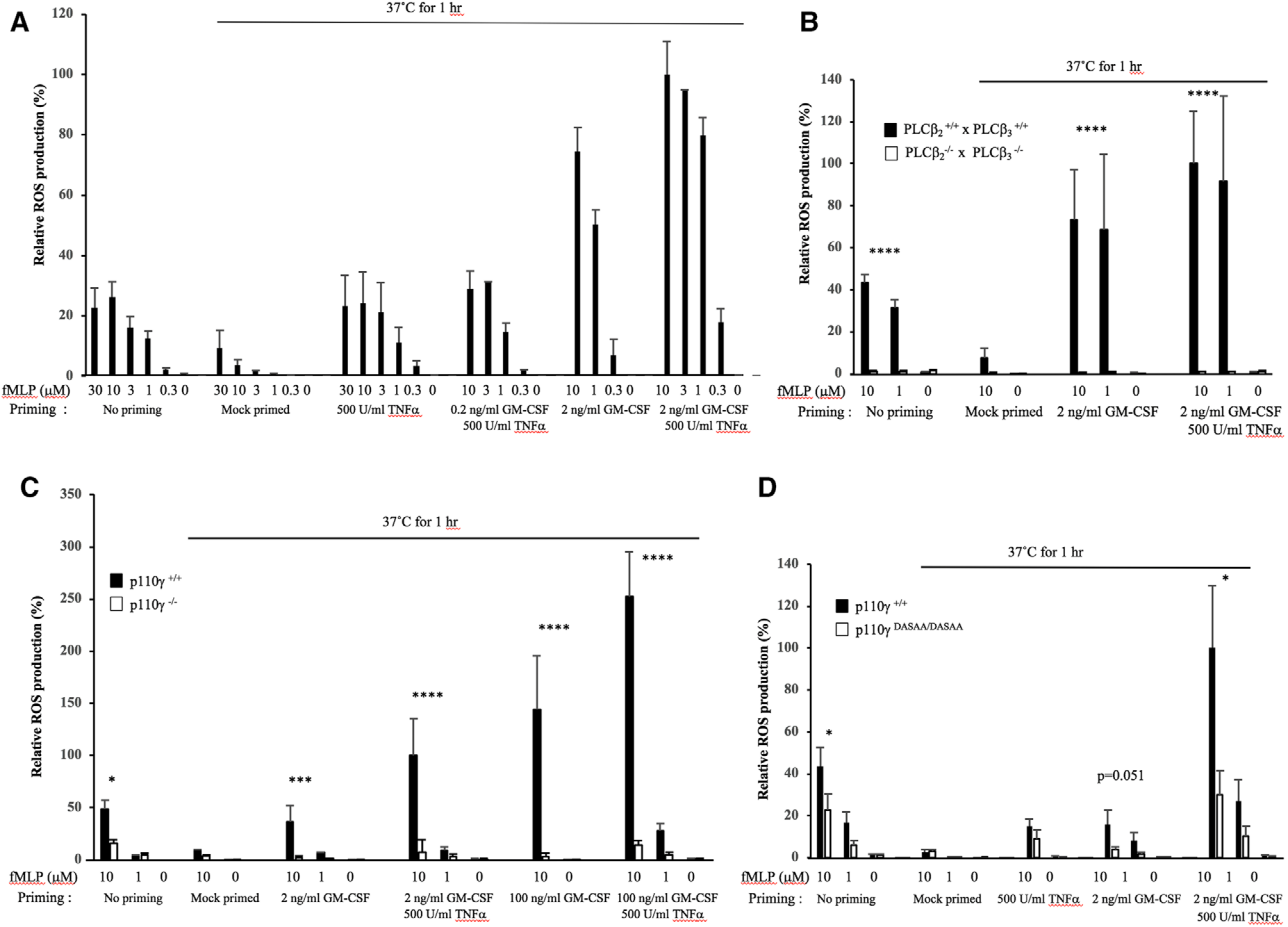
**Contribution of PLCβ and PI3Kγ pathways to ROS production by unprimed and primed mouse neutrophils**. (A) ROS production was recorded in WT mouse neutrophils. Data shown are the peak ROS levels with 100% representing the peak response obtained with 10 µM fMLP in cells primed with 2 ng/ml GM‐CSF and 500 U/ml TNF‐α. The data show that 2 ng/ml, but not 0.2 ng/ml, GM‐CSF synergizes with TNF‐α (also see Fig. 2A). The data are presented as mean ± sem of 3–8 independent experiments performed in duplicate, except for the following conditions: 3 µM fMLP for 0.2 ng/ml GM‐CSF/500 U/ml TNF‐α and 3 µM fMLP for 2 ng/ml GM‐CSF/500 U/ml TNF‐α, where *n* = 1. (B) ROS production from neutrophils isolated from PLCβ_2_^+/+^ x PLCβ_3_^+/+^ and PLCβ_2_^−/−^ x PLCβ_3_^−/−^ mice. The data are presented as peak ROS levels with 100% representing the peak response obtained with 10 µM fMLP in PLCβ_2_^+/+^ x PLCβ_3_^+/+^ cells primed with 2 ng/ml GM‐CSF and 500 U/ml TNF‐α. The data are presented as mean ± sem of 3–6 independent experiments performed in duplicate, except for the mock‐primed condition with the PLCβ_2_^−/−^ x PLCβ_3_^−/−^ neutrophils, 10 µM and 0 µM fMLP, where means ± range of *n* = 2 independent experiments are shown. (C) ROS production from neutrophils isolated from p110γ^+/+ ^and p110γ^–/–^ mice. Data are presented as peak ROS levels with 100% representing the peak response obtained with 10 µM fMLP in p110‐γ^+/+ ^ neutrophils primed with 2 ng/ml GM‐CSF and 500 U/ml TNF‐α and are presented as mean ± sem of 3–8 independent experiments. (D) ROS production from neutrophils isolated from p110‐γ^+/+^ and p110γ^DASAA/DASAA^ mice. Data are presented as peak ROS level with 100% representing the peak response obtained with 10 µM fMLP in p110‐γ^+/+ ^neutrophils primed with 2 ng/ml GM‐CSF and 500 U/ml TNF‐α and are presented as mean ± sem 3–8 independent experiments performed in duplicate, except for the 500 U/ml TNF‐α results, which are means ± range of *n* = 2. Significance of the differences was estimated using unpaired ANNOVA test. *P ≤ 0.050020 vs. WT mice, **P ≤ 0.01 vs. WT mice, ***P ≤ 0.0005 vs. WT mice, ****P ≤ 0.0001 vs. WT mice

**Figure 2:**
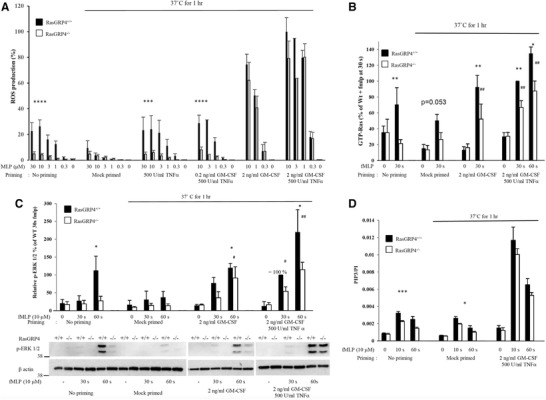
**RasGRP4 has a more important role in unprimed neutrophils**. (A) ROS production from neutrophils isolated from RasGRP4^+/+^ or RasGRP4^–/–^ mice. Data are presented as mean ± sem 3–8 independent experiments performed in duplicate except for the conditions 3 µM fMLP for 0.2 ng/ml GM‐CSF/500 U/ml TNF‐α and 3 µM fMLP for 2 ng/ml GM‐CSF/500 U/ml TNF‐α, where *n* = 1. (B) Quantification of GTP‐Ras measured by affinity pull‐down with GST‐RafRBD. The data were normalized to the amount of β‐actin as a loading control. Data are presented as mean ± sem of 3–9 independent experiments. (C) Neutrophil lysates were analyzed by Western blots for pERK1/2 (anti‐phospho‐T202/Y204) and β‐actin. Top: quantification of the gels. The data are presented as mean ± sem of 3–4 independent experiments. Bottom: representative immunoblot. (D) PIP3 analysis from RasGRP4^+/+^ and RasGRP4^−/−^ mouse neutrophils. The data presented are means ± sem of 3 independent experiments performed in duplicate. Significance of the differences was estimated using unpaired ANNOVA test. *P ≤ 0.05 vs. WT mice, **P ≤ 0.01 vs. WT mice, ***P ≤ 0.0005 vs. WT mice, ****P ≤ 0.0001 vs. WT mice. ^#^ *P* ≤ 0.05 vs. RasGRP4^−/−^ mice at *t* = 0, ^##^ *P* ≤ 0.01 vs. RasGRP4^−/−^ mice at *t* = 0

Incubating freshly isolated mouse neutrophils at 37°C for 1 h in the absence of priming agents leads to a 2–3‐fold reduction, compared to freshly prepared cells, in the amount of ROS they produce in response to fMLP (Fig. 1). The reason for this decline in responsiveness is unclear but has been observed previously.^24^ Quite surprisingly, there was no parallel reduction in fMLP‐stimulated PIP_3_ accumulation (see Fig. 2D). When mouse neutrophils were primed for 1 h with GM‐CSF and TNF‐α (2 ng/ml and 500 U/ml), there was about a 10‐fold increase, compared to mock‐primed cells, in the amount of ROS generated in response to fMLP; however, priming had no effect on ROS production in the absence of fMLP (Fig. 1). Treatment with either TNF‐α or GM‐CSF alone also primed fMLP‐stimulated, but not basal, ROS formation, by relatively smaller extents (Fig. 1A). Mouse neutrophils lacking both PLCβ2 and PLCβ3 failed to produce ROS in response to fMLP, either in the absence (confirming previous results) or presence of priming agents (Fig. 1B). These results are consistent with the idea that PLCβ2/β3‐dependent changes in cytosolic free Ca^2+^ and DAG/PKC are required for GPCR‐stimulated ROS formation^7^ and that priming does not change this dependency. Mouse neutrophils lacking p110‐γ produced substantially less ROS in response to fMLP in both unprimed or TNF‐α‐ and GM‐CSF‐primed conditions (Fig. 1C), confirming previous work^24^ and consistent with the central role of PI3K‐γ in GPCR stimulation of ROS formation.^7^, ^8^, ^9^ Mouse neutrophils expressing an endogenous, Ras‐insensitive version of p110‐γ (p110γ^DASAA/DASAA^) had a similar, but weaker, phenotype compared to p110γ^–/–^ neutrophils (Fig. 1D). This result demonstrates that a Ras input to PI3K‐γ is needed to allow normal fMLP‐stimulated ROS formation in both unprimed^15^ and primed neutrophils.

### 3.2 RasGRP4 is required for fMLP‐stimulated ROS formation, PIP_3_ accumulation, and both Ras and ERK activation in unprimed neutrophils, but has a much reduced role in primed cells

Unprimed neutrophils lacking RasGRP4 displayed substantially reduced ROS production (Fig. 2A), Ras activation (Fig. 2B), ERK phosphorylation (Fig. 2C), and PIP_3_ accumulation (Fig. 2D), in response to fMLP, compared to WT cells, consistent with previous results.^16^ In contrast to these results and to those obtained with p110γ^–/–^, PLCβ2/β3‐double‐KO, and p110γ^DASAA/DASAA^ neutrophils, primed RasGRP4^–/–^ neutrophils had indistinguishable fMLP‐stimulated ROS formation (Fig. 2A) and PIP_3_ accumulation (Fig. 2D), and retained substantial fMLP‐stimulated increases in Ras and ERK activation (Fig. 2B and C), compared to WT controls. These results suggest that although RasGRP4 has a unique role, or limits fMLP responses in unprimed neutrophils, additional mechanisms are used by fMLP in primed cells to activate Ras, and through this, PIP_3_, ERK signaling, and ROS formation. These additional mechanisms are dominant over RasGRP4 in TNF‐α‐ and GM‐CSF‐primed cells. It is also clear that the priming agents themselves (TNF‐α and GM‐CSF, or GM‐CSF alone) do not stimulate these new mechanisms leading to activation of Ras.

### 3.3 SOS1/2 become the functionally dominant, fMLP‐sensitive RasGEFs in TNF‐α‐ and GM‐CSF‐primed neutrophils

**Figure 3:**
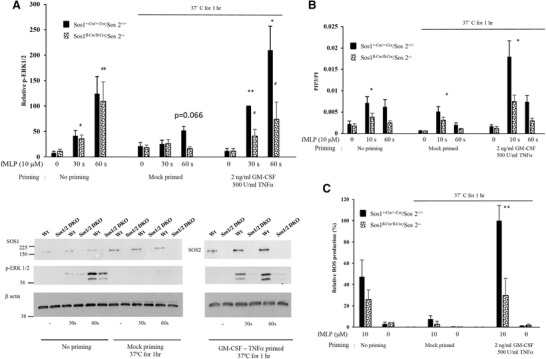
**SOS 1/2 is only essential in primed neutrophils**. (A) Neutrophils lysates were analyzed by Western blots for pERK1/2 (anti‐phospho‐T202/Y204). Bottom: a representative immunoblot of pERK1/2, SOS1, SOS2, and β‐actin. Gels were quantified and data presented as mean ± sem, *n* = 3–4 independent experiments, except for the mock‐primed data, where means ± range of *n* = 2 are shown. (B) PIP3 analysis from neutrophils from SOS1^+/cre/+/cre^ /SOS2^+/+^ or SOS1^fl/cre/fl/cre^ /SOS2^–/–^ mice. The data presented are presented as mean ± SEM from 3–6 independent experiments performed in duplicate. (C) ROS production from neutrophils from either SOS1^+/cre/+/cre^ /SOS2^+/+^ or SOS1^fl/cre/fl/cre^ /SOS2^‐/−^ mice. The data are presented as mean ± sem of 3–4 independent experiments performed in duplicate, except for the conditions with SOS1^fl/cre/fl/cre^ /SOS2^–/–^ neutrophils stimulated with 10 µM fMLP in the mock‐primed condition, where means ± range of *n* = 2 are shown. Significance of the differences was estimated using unpaired ANNOVA test. *P ≤ 0.05 vs. WT mice, **P ≤ 0.01 vs. WT mice, ***P ≤ 0.0005 vs. WT mice, ****P ≤ 0.0001 vs. WT mice. ^#^ *P* ≤ 0.05 vs. SOS1^fl‐Cre/fl‐Cre^/SOS2^–/–^ mice at *t* = 0, ^##^ *P* ≤ 0.01 vs. SOS1^fl‐Cre/fl‐Cre^/SOS2^–/–^ mice at *t* = 0

We fed SOS2^−/−^ x SOS1^LoxP/LoxP^ x ERT2‐Cre mice a diet containing tamoxifen (see the Material and Methods Section), yielding “SOS1/2‐DKO” mice, prepared neutrophils, lysed them, and immunoblotted for SOS1 and 2 (Fig. 3A). This revealed the neutrophils lacked detectable SOS1 or 2. However, we could only recover approximately 2 × 10^6^ neutrophils/mouse from these mice, about 20–25% of the yield from control mice indicating that SOS1/2 may have an important role in differentiation or proliferation of neutrophil progenitors. In fact, a previous report showed that other bone marrow progenitors such as lymphocyte progenitors were also affected by SOS1/2 depletion and that the peripherical neutrophils number was also down.^25^

The low recovery neutrophils number has severely restricted the experiments we could conduct; thus, direct assays of Ras activation were impractical. It has been shown previously, however, that in fibroblasts isolated from the same mouse strains, loss of SOS1/2 resulted in an almost complete blockade of EGF or PDGF‐stimulated Ras activation.^28^ In unprimed cells, fMLP‐stimulated ERK activation (Fig. 3A) and ROS formation (Fig. 3C) were not significantly altered, whereas PIP_3_ accumulation was reduced modestly, by the loss of SOS1 and 2. In contrast, in TNF‐α‐ and GM‐CSF‐primed neutrophils, all three fMLP‐induced responses were substantially reduced in SOS1/2‐DKO cells. These results indicate that priming with TNF‐α and GM‐CSF has made SOS1/2 more sensitive to acute stimulation with fMLP, leading to greater SOS‐dependent activation of Ras. We considered the possibility that priming for 1 h with TNF‐α and GM‐CSF had increased the amount of SOS1 and/or 2 in the neutrophils. SOS 1 and 2 were quantified in comparison to a loading control by immunoblotting. This revealed that priming with TNF‐α and GM‐CSF did not significantly change the levels of SOS 1 or 2 proteins (see the Supplementary Information).

## 4 CONCLUDING REMARKS

Our results build on work published by other labs identifying roles for a Ras‐sensitive‐network controlling important neutrophil functions such as ROS formation, NET release,^29^ and tissue infiltration (in acute pancreatitis^30^) and in disease processes, such as autoimmune vasculitis (via antineutrophil cytoplasmic antibodies—ANCA^31^). Our results provide a molecular framework with which to understand how Ras signaling orchestrates ROS formation in basal and primed neutrophils.

The results presented above confirm that in neutrophils, the primary class I PI3K required for a wide variety of GPCRs to stimulate ROS formation is PI3K‐γ and that Ras‐family GTPases play a key role in its activation.^15^, ^24^ These conclusions contrast with studies of macrophage‐like bone marrow‐derived cells that show that PI3K‐δ is the key player in Ras signaling networks constitutively activated by mutations in PTPN11 (Shp2) common in juvenile myelomonocytic leukaemia.^32^

Collectively, our results indicate that the level of Ras activation achieved in primed cells via SOS is sufficient to support enhanced class I PI3K activation, ERK activation, and ROS formation, without a requirement for RasGRP4. This may be a result of the ability of GTP‐bound Ras to stimulate SOS proteins, and hence reinforce activation of Ras through a “feedforward loop”^18^; however, in this scenario, it is clear that priming would be required for fMLP to drive SOS1/2 to a threshold of positive reinforcement. The implication of these results is that SOS proteins are important determinants of the responsiveness of primed, but not unprimed neutrophils. These signaling mechanisms augment the scale of potentially damaging neutrophil responses specifically in zones of inflammation but retain a requirement for acute activation by a coincident proinflammatory GPCR‐binding mediator and hence act to restrict potential collateral tissue damage. These mechanisms leading to an important role for SOS‐family RasGEFs are almost certainly active in TANs; however, their impact on tumor progression remains unclear and will be a function of their significance in either tumor‐supporting or tumor‐suppressive roles of the different subtypes of neutrophils.^33^

## AUTHORSHIP

S.S. and K.D. performed experiments; A.S.‐P. performed statistical analysis; F.B. and E.S. supplied the SOS1^fl‐Cre/fl‐Cre^/SOS 2^−/−^ mice and their control (SOS1^+‐Cre/+‐Cre^/SOS 2^+/+^); S.S., P.T.H., and L.R.S. designed research, analyzed data, and wrote the article. P.T.H. and L.R.S. contributed equally to this work.

## DISCLOSURES

The authors declare no conflicts of interest.

## Supporting information

Supplementary Figure 1
Phorbol myristate acetate (PMA)‐driven ROS production is not affected by the genetic manipulations examined in this study.
