# Supplementary material for: Frontline Science: TNF‐α and GM‐CSF1 priming augments the role of SOS1/2 in driving activation of Ras, PI3K‐γ, and neutrophil proinflammatory responses
Source: J Leukoc Biol. 2019 Feb 5;106(4):815–22. doi: 10.1002/JLB.2HI0918-359RR (PMC6977543; doi:10.1002/JLB.2HI0918-359RR)
Supplement: Supplementary file 1 — Supplementary Figure 1 Phorbol myristate acetate (PMA)‐driven ROS production is not affected by the genetic manipulations examined in this study. [file JLB-106-815-s001.pdf]

Supplementary Figure 1

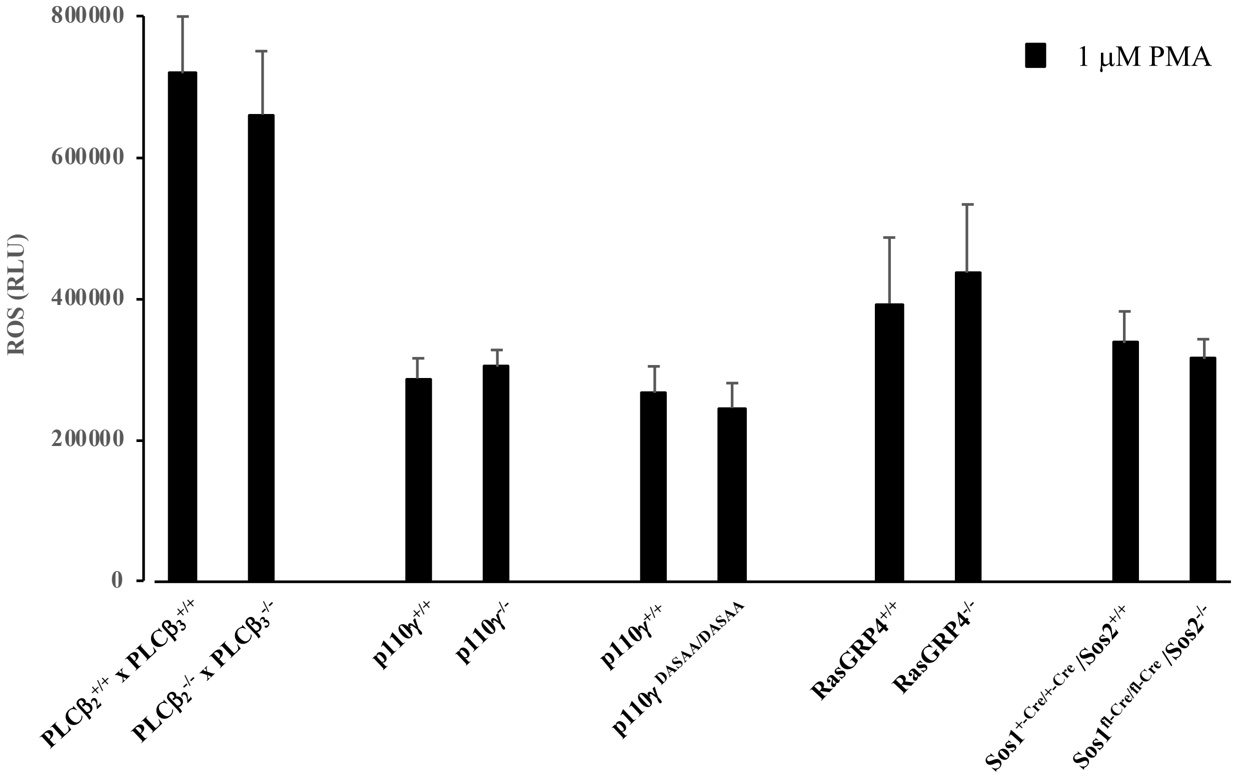

Phorbol myristate acetate (PMA)-driven ROS production is not affected by the genetic manipulations examined in this study. ROS production from neutrophils isolated from $PLC\beta_2^{-/-}$ x $PLC\beta_3^{-/-}$, $p110\boldsymbol{\gamma}^{-/-}$, $p110\boldsymbol{\gamma}^{DASAA/DASAA}$ $RasGRP_4^{-/-}$ and $SOS1^{fl/cre/fl/cre}$ /$SOS2^{-/-}$ mice and their respective 'Wt strain' controls. Data are presented as mean ± SEM of 3-8 independent experiments performed in duplicate, except for the RasGRP4 and $SOS1^{-/cre/-/cre}$/$SOS2^{-/-}$ conditions, where the data are means ± range of n=2. No significant differences were observed within the same strain background.

Supplementary Figure 2

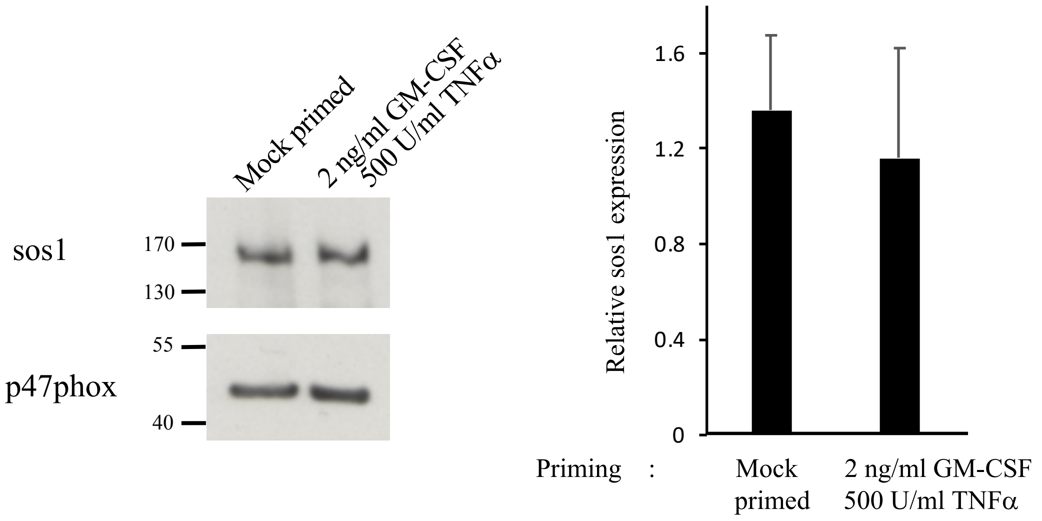

SOS1 expression is not affected by priming.
WT mouse neutrophil lysates were analysed by Western blots for SOS1 expression under mock-primed and primed conditions (1 hr at 37ºC, 2 ng/ml GM-CSF/500 U/ml TNFα). Data are presented as mean ± SEM of 4 independent experiments and the data were normalized with the loading control p47phox.
